# A Dyadic Pain Management Program for Community-Dwelling Older Adults with Chronic Pain: Study Protocol for a Cluster Randomized Controlled Trial

**DOI:** 10.3390/ijerph191912186

**Published:** 2022-09-26

**Authors:** Mimi M. Y. Tse, Shamay S. M. Ng, Vivian Lou, Raymond Lo, Daphne Sze Ki Cheung, Paul Lee, Angel S. K. Tang

**Affiliations:** 1 School of Nursing and Health Studies, Hong Kong Metropolitan University, Hong Kong, China; 2 Department of Rehabilitation Sciences, The Hong Kong Polytechnic University, Hong Kong, China; 3 Department of Social Work and Social Administration, The University of Hong Kong, Hong Kong, China; 4 Geriatrics and Palliative Medicine, Shatin Hospital, Hong Kong, China; 5 School of Nursing, The Hong Kong Polytechnic University, Hong Kong, China; 6 Department of Health Sciences, University of Leicester, Leicester LE1 7RH, UK; 7 School of Nursing, Caritas Medical Centre, Hospital Authority, Hong Kong, China

**Keywords:** dyadic pain management, community-dwelling older adults, chronic pain, randomized controlled trial

## Abstract

Community-dwelling older adults suffer from chronic pain. Pain negatively affects their physical and psychosocial wellbeing. The majority of pain management education and programs focus only on older adults. Their informal caregivers should be involved in pain management. A dyadic pain management program for reducing pain and psychological health symptoms, and improving pain self-efficacy, quality of life, and physical function in older adults is proposed for evaluation of its effectiveness. This will be a cluster randomized controlled trial. Community-dwelling older adults aged 60 or above and their informal caregivers will be recruited. The dyadic pain management program will be an eight-week group-based program. The participants in the experimental group will receive four weeks of center-based, face-to-face activities and four weeks of digital-based activities via a WhatsApp group. The control group will receive the usual care and a pain management pamphlet. Data will be collected at baseline, and at the eighth-week and sixteenth-week follow-up session. The outcome measurements will include pain intensity, pain self-efficacy, perceived quality of life, depression, anxiety, and stress levels. Data on the caregiver burden will be collected from the informal caregivers. Because of the COVID-19 pandemic, all social activities have been suspended. In the near future, as the pandemic subsides, the dyadic pain management program will be launched to benefit community-dwelling older adults and informal caregivers and to reduce their pain and the care burden, respectively.

## 1. Introduction

The prevalence of chronic non-cancer pain is as high as 37% among community-dwelling older adults [1,2]. Pain is associated with significant physical and psychosocial incapacities [1,2,3]. Older adults with pain are more depressed and anxious and have reduced social interaction compared to those without pain [1,2,3]. Pain in older adults tends to be constant in nature, moderate to severe in intensity, and years long in duration [3,4]. Due to the physical vulnerability and immobility caused by chronic pain, older adults have decreased self-care ability and are at a high risk of falls [3,4].

Pain is often inadequately managed [4,5]. Analgesics remain the primary approach to managing pain [4,5]. However, older adults worry about adverse drug reactions and accept chronic pain as part of aging [2,3,4]. Hence, non-pharmacological strategies for managing pain, including pain education programs, exercise, massage, relaxation, and listening to music, are becoming increasingly popular [4,5,6].

Ersek et al. developed a pain management program using pain education, drugs, and a variety of non-pharmacological techniques, and found significant reductions in pain and improvements in physical and psychological parameters among the experimental group of older adults [7,8]. Drawing on their work, Tse et al. [9] implemented a pain management program that involved recruiting and training older adults to serve as peer volunteers in leading a pain management program for older adults living in nursing homes. After completing the program, the older adults were happier and reported less pain.

However, our peer-led pain management model may not benefit community-dwelling older adults with chronic pain, who require constant care from family members and caregivers [10]. In Hong Kong, more than 90% of older adults were found to be living in domestic households; of those, 25.2% were living only with their spouse, 29.0% were living with a spouse and children, and 19.5% were living only with their children [11]. That means that older adults are mainly being cared for by informal caregivers. An informal caregiver is a family member or close friend who has taken responsibility for the physical and emotional needs of a person who cannot entirely care for himself or herself because of advanced age, illness, dementia, or disability without an income [11].

Informal caregivers of older adults with chronic pain have a wide range of responsibilities that normally include helping the elderly in activities of daily living, reminding them to take their medications and to do exercises to relieve pain symptoms, taking them to the doctor when necessary, communicating with them, providing emotional support, and encouraging them to engage in social activities [12]. Caring responsibilities include providing help with daily activities and exercises to relieve pain symptoms and communicating with the older adults and providing them with emotional support [10,13]. Family members and carers have stated that it can be difficult to communicate with older adults with chronic non-cancer pain or to encourage them to participate in various non-pharmacological strategies for managing pain [8,9].

The majority of existing services focus on offering pain management education and programs to older adults/clients only, instead of to both older adults and their caregivers. Family members are important sources of interpersonal influence that can increase or decrease an individual’s commitment to engaging in health-promoting behavior [13,14]. Therefore, pain management programs should pair an older adult with his/her caregiver as a “dyad”.

Notably, the Health Promotion Model (HPM) describes the multidimensional nature of persons interacting with their interpersonal and physical environments in connection with the issue of health [13,14]. Utilizing the HPM, interventions involving a certain type of social support, such as a “dyadic” system, have a greater potential to increase the participation of older adults, encourage greater adherence to health-promoting activities, and result in a longer term of commitment and higher level of enjoyment [13,14].

A dyadic intervention could encourage patients to constantly take part in the intervention and enable their caregivers to be included in the same intervention. Dyadic interventions have been examined for clients with depression and dementia, yet, to the best of our knowledge, only two studies have used patient/caregiver dyads in a pain management intervention [15,16]. Keefe et al.’s [15] study targeted adults (mean age of 57) with osteoarthritic knee pain. Abbasi et al.’s [16] study targeted adult patients (aged 24–67 years) with chronic low back pain. Both studies involved pain-coping skills and exercise training and demonstrated improvements in pain intensity and psychological distress.

However, both studies were applied to those with osteoarthritic knee pain [15] or chronic low back pain [16]. In both studies, no prior power analysis had been conducted to estimate the sample size, putting their validity in doubt. Lastly, Abbasi’s study involved a multi-disciplinary team and intensive support from clinical psychologists, which could mean that the intervention might not be an affordable solution for patients with pain. More evidence is needed to determine whether the findings are generalizable to patients with other types of pain. It is for this reason that the present study is proposed.

## 2. Materials and Methods

### Aim and Objectives

To evaluate the effectiveness of a dyadic pain management program (DPM) in reducing pain and psychological health symptoms and improving pain self-efficacy, quality of life, and physical function in older adults when compared with a control population that received the usual care.

Primary objective:

To evaluate the effectiveness of a DPM in reducing pain scores.

Secondary objectives:

To evaluate the effectiveness of a DPM in reducing pain interference and psychological health symptoms (including levels of depression, anxiety, and stress), and enhancing pain self-efficacy and quality of life.

To explore the perspectives and experiences of informal caregivers and older adults on participating in the DPM.

## 3. Design and Setting of the Study

A cluster randomized controlled trial (RCT) will be used. It is a superior trial. Our study will be conducted in Neighborhood Elderly Centers (NECs) subsidized/run by the Social Welfare Department of Hong Kong. NECs offer a comprehensive range of community support services at the neighborhood level to enable older adults to remain in the community; lead a healthy, respectable, and dignified life; and contribute to society.

The target groups for NECs are adults aged 60 or above who live in the locality, carers, and the community at large. The scope of an NEC’s services include: providing health education, educational and developmental activities, information on community resources and referral services, volunteer development programs, carer support services, educational and support programes on dementia, counselling services, outreach and networking services, social and recreational activities, meal services, drop-in services, and so on. There are 169 NECs throughout the 18 districts in Hong Kong that are highly accessible to older adults in the community [17].

We chose NECs for collaboration because the major scope of their services is health promotion and carer support services, and because their convenient location in residential areas means that they are accessible to potential participants. The purpose of our research is in line with that of the NECs, which is to enable community-dwelling senior citizens to lead healthy and purposeful lives. Letters will be sent to all 169 NECs, inviting them to join the study. Those interested in participating will be randomized into the experimental or control group according to a computer-generated list. The unit of randomization is the NEC. The randomization will take place at the beginning of the study, and each NEC will be randomized into either an experimental group or a control group. This will prevent any possible contamination effect.

Allocation concealment, blinding, and the control arm: a statistician independent of the study team will use a random numbers table to make the assignments (1 = experimental DPM; 2 = control). The experimental group will receive the DPM and the control group will receive the usual care and a pain management pamphlet.

## 4. Sample Size Estimation and Justifications

No similar study has been conducted thus far. In our pilot study, both groups showed a significant reduction in pain scores with large effect sizes (Cohen’s d > 0.9 for the intervention group). There was no change in the pain score of the control group. Thus, the sample size was estimated based on the pain score of the older adults in our pilot study [18]. Using a significance level of 5% and a power of 80%, we arrived at a total estimated sample size of 150 dyads (one older adult and his/her caregiver as one dyad) with an effect size of 0.762 (=1.6/2.1) on the pain score, taking into consideration a 20% drop-out rate and a 0.1% intra-cluster correlation (from a review of 31 cluster-based studies in primary care) [19]. Thus, there will be 75 dyads for the experimental group and 75 dyads for the control group.

We expect to recruit 150 dyads from 20 NECs. A dyad consists of an older adult and their informal caregiver. Based on our pilot study [15] and our previous studies in NECs [2], an average of around 7 dyads can be recruited in an NEC. Therefore, 22 NEC clusters (12 experimental groups and 12 control groups) will be required for the target sample.

## 5. Inclusion and Exclusion Criteria

Older adults/Participants: Inclusion criteria.

- Aged 60 or above and being cared for mainly by an informal caregiver [11], with both the older adult and carer willing to participate in the DPM together.
- Scored > 6 in the Abbreviated Mental Test; a cut-off point of 6 is valid for differentiating between normal and abnormal cognitive functions in geriatric clients [10].
- Can understand Cantonese.
- Has a history of non-cancer pain in the past 6 months [20].
- Has a pain score of at least 2 on the Numeric Rating Scale (0–11 numeric scale) [21].
- Able to take part in light exercise and stretching.
- One member of the dyad owns a smartphone and can access the Internet.

Older adults/Participants: Exclusion criteria.

- Has a serious organic disease or malignant tumor.
- Has a mental disorder diagnosed by neurologists or psychiatrists.
- Will have further medical/surgical treatment in two months.
- Experienced drug addiction [22].

Informal Caregivers: Inclusion criteria.

- Aged 18 or above.
- Acting as an informal caregiver [11] for the participating older adult.
- Scored > 6 in the Abbreviated Mental Test; a cut-off point of 6 is valid for differentiating between normal and abnormal cognitive functions in geriatric clients [10].
- Can understand Chinese.
- Has a history of non-cancer pain in the past 6 months [20].
- Has a pain score of at least 2 on the Numeric Rating Scale (0–11 numeric scale) [21].
- Able to take part in light exercise and stretching.
- Owns a smartphone and can access the Internet.
- Able to attend whole sessions in the community activity center.

Informal Caregivers: Exclusion criteria.

- Has a serious organic disease or malignant tumor.
- Has a history of cognitive or mental disorder diagnosed by neurologists or psychiatrists.
- Will have further medical/surgical treatment in two months or have joined another pain management program.
- Experienced drug addiction [20].

## 6. Intervention—Dyadic Pain Management Program (DPM)

The dyadic pain management program (DPM): The DPM is an 8-week group-based program. The DPM is comprised of 4 weeks of center-based, face-to-face activities and 4 weeks of digital-based activities delivered via a WhatsApp group. An 80% participation rate in the face-to-face activities will be regarded as completion of the DPM [9]. Timely make-up sessions will be arranged for those unable to attend the scheduled session. The experience from our pilot study indicates that there will be around 6–7 dyads in each DPM [18].

With regard to the face-to-face part, the DPM will start with 20–30 min of physical exercise supervised by the research assistant, followed by 20 min of pain management education, including information on the impacts of pain, the use of drug and non-drug strategies for pain management, and demonstrations and return demonstrations of various non-drug pain management techniques. Communication skills regarding the practice of various pain management techniques by the participants and their caregivers will be taught in the face-to-face session, and the participants will be encouraged to practice various pain relief methods at home. At the end of the session, the caregiver and research assistant will help the older adults to make portfolio entries on the activities of the day, to help them recall the various pain relief methods learned in each class [9].

For the home-based part, an exercise book will be given to each dyad to guide them in performing exercises at home. The recommendation is that they perform 30 min of exercise, three times a week, at home, but the more frequently, the better. The research assistant will show them how to use this exercise book in week 1 of the face-to-face sessions. The exercise book contains detailed images of each step in the exercises, which are the same exercises as those performed by the exercise dyads in the community center. This book will be given to each dyad to ensure that the participants know how to perform the exercises at home. The exercise book includes information on the whole exercise process: warming up, breathing, stretching, strength exercises, balance, and relaxation exercises. Reminders to practice and to record the completion rate will be sent via WhatsApp.

The use of a WhatsApp group (digital-based activities): All participants will join a WhatsApp group to receive teaching materials and videos of the physical exercises learned in class, for practice at home. Each dyad will be encouraged and reminded to practice the 30 min exercises together, three times per week, at home, and make entries in the WhatsApp group; and also to record their use of various types of non-pharmacological methods to relieve pain and their perception of the effectiveness of those methods. The team will produce a CD with video clips of the exercise for the dyads if the exercise video clips are too large to download from the WhatsApp group.

Protocols that govern the implementation of the planned measures and that act as a quality assurance mechanism.

### 6.1. Protocol for Carrying out the DPM Intervention (Intervention Fidelity)

The DPM protocol would include: (1) the goal of the DPM; (2) special contents and activities of each DPM and specific instructions for each session. This protocol will be read and signed by the research assistant (for the experimental group) before each DPM session to ensure consistency.

To optimize fidelity, several strategies will be used. First, the research assistant will train the research team to lead the DPM. Second, a detailed teaching manual and questions for digital-based activities were developed and tested. Third, the research assistant will participate in biweekly meetings with the research team to discuss and review cases and receive “booster” sessions on pain management education. Fourth, a process evaluation, fidelity checklist, and semi-structured interview will be employed.

### 6.2. Protocol for the Control Group

The participants in the control group will receive the usual care and a pain management pamphlet. We believe that reading the pamphlet could help the participants to manage their pain but would be less efficacious than the DPM.

### 6.3. Protocol for the Preparation of NECs and the Collaboration Plan

The face-to-face sessions will be carried out in a multi-function room/larger room, preferably with comfortable chairs, adequate lightning, and good ventilation. NEC staff will ensure that the older adults and their caregivers are wearing comfortable clothing when participating. Additionally, the research team will alert the NEC staff and work with them to ensure environmental safety, reduce fall risks, and improve the mobility of all participants. In light of the COVID-19 situation, we would introduce modifications if necessary, including replacing the center-based activities with sessions held over Zoom/online-based contact. This would be possible because the informal carers would have a smart phone with access to the Internet. The research team, with the help of the NEC staff, can contact the informal carer via phone and link up to Zoom for center-based activities and further actions.

## 7. Data Collection and Outcome Measures

A 36-month timeline is essential to recruit the required sample. Data collection will be carried out simultaneously in multiple sites.

Data will be collected at three time points: At baseline (T0), week 8 (T1), and week 16 (T2), using standardized methods and questionnaires, with a follow-up assessment (T2) to determine whether the observed benefits can be sustained over a longer period of time.

### 7.1. Primary Outcome

1. Pain intensity:

The Chinese version of the Brief Pain Inventory will be used to assess the multidimensional nature of pain, including intensity and interference with life activities in the previous 24 h [22]. Coefficient alphas for the pain severity and pain interference items were 0.894 and 0.915, respectively [22].

### 7.2. Secondary Outcomes

2. Pain self-efficacy:

A Chinese version of the Pain Self-Efficacy Questionnaire (PSEQ) will be used to measure self-efficacy in coping with activities despite pain [23]. It consists of 10 statements about a person’s confidence in performing 10 activities or tasks despite experiencing pain. Higher scores indicate stronger self-efficacy beliefs. The test–retest reliability coefficient was 0.75, and the Cronbach’s alpha for internal consistency was 0.95, showing that the tool is reliable [23].

3. Perceived quality of life:

The Chinese version of the EQ-5D-5L [24] will be used to measure the quality of life of the participants as well as the cost effectiveness of the proposed dyadic pain management program. EQ-5D-5L is a health-related quality-of-life measure developed by the EuroQol Group. It is based on a descriptive system that defines health in terms of five dimensions: Mobility, Self-Care, Usual Activities, Pain/Discomfort, and Anxiety/Depression. Each dimension has three response categories corresponding to no problems, some problems, and extreme problems. EQ-5D-5L has been widely tested and used in the general population and patient samples and has been translated into over 130 different languages.

The EQ-5D-5L has been validated and used in the Hong Kong context in clinical and cost-effectiveness studies. A comparison of scores (e.g., a value set from 1 (full health) to 0 (dead)) reflects an estimation of quality-adjusted life years (QALYs) before and after the intervention; with reference to the Hong Kong norm, this would provide evidence of the effectiveness and cost-effectiveness of the proposed dyadic pain management program for community-dwelling older adults with chronic pain.

4. Psychological health: Depression, anxiety, and stress:

The Depression Anxiety Stress Scales 21-items (DASS-21) is a self-administered psychological instrument to evaluate degrees of depression, anxiety, and stress [25]. Every part has seven items on a 4-point Likert scale ranging from 0 to 3. The Cronbach’s α was 0.912 and the test–retest Pearson correlation coefficient was 0.751 [25].

5. Caregiver burden inventory (for the caregivers only):

The Caregiver Burden Inventory [26] comprises 24 items measuring five dimensions of burden related to the caregiving role. These are: (1) time-dependence; (2) developmental burden; (3) physical burden; (4) social burden; and (5) emotional burden. Participants are asked how often each statement describes their feelings on a scale ranging from 0 (never) to 4 (nearly always). Alpha coefficients for each subscale were satisfactory and ranged from 0.74 to 0.88. The overall internal consistency was a = 0.91.

6. Process evaluation:

A process evaluation will be carried out to identify the strengths and limitations of the intervention from the perspectives of the older adults and their caregivers. The results will help the research team to understand the therapeutic features of the DPM and the barriers and difficulties faced by the participants. This will increase our understanding of the processes underlying the effects of the intervention and explain how it works best in practice. The timing and methods of data collection will include:

Field observations:

These will be carried out in the NECs to monitor the quality of the implementation of the DPM. Each DPM will be observed twice (by random selection among the four sessions) when carrying out the DPM. A total of 48 observations will be conducted by the principal investigator, guided by a fidelity checklist. The fidelity checklist was developed based on a similar intervention study for chronic pain [9]. The checklist indicates the implementation of the DPM in terms of four levels: low/not observed; observed to a small degree; observed to a medium degree; and high implementation. To ensure quality and consistency, the research assistant should demonstrate 95% implementation at a high level [23]. The compliance rate will be calculated. Field notes will also be taken on outstanding events. The data will be examined to determine whether the research assistant carried out the implementation according to the teaching manual and protocol.

Knowledge and skills acquired in managing pain situations for older adults:

A questionnaire will be given to the participants in the experimental and control groups regarding their knowledge and skills in managing pain situations at baseline (T0), week 8 (T1), and week 16 (T2).

Semi-structured interviews for older adults and their caregivers:

Individual interviews will be conducted by the RA1 within 2 weeks after T1 (post-test). We will recruit 8 older adults and their caregivers (20%) from the experimental group and 8 (20%) from the control group, or until data saturation is reached. To obtain the widest range of opinions and comments from different perspectives, equal proportions of participants (1) with significant positive changes between the baseline and first post-test measurements, (2) without significant changes, and (3) with negative changes will be invited for interviews. They will be asked to comment on their experiences and feelings about the intervention that they received and on changes in their behavior; their perceptions of the intervention; their beliefs, concerns, and views of the difficulties of managing pain; and on how the intervention could be improved to meet their needs.

A semi-structured interview guide was developed for interviews in Cantonese. Participants will be asked about their experiences in joining the DPM; the perceived benefits, limitations, and barriers to pain management; its usefulness; and recommendations for improving the DPM.

A SPIRIT figure is used to illustrate the timepoints for enrolment, interventions, and assessment (Table 1) [27].

## 8. Results Analysis

IBM-SPSS version 22 will be used to perform statistical analyses. One member of the research team is a statistician and will discuss the data analysis with the team. Descriptive statistics (frequency%; mean (standard deviation)) will be used to describe the demographic data of the participants.

An intention-to-treat analysis will be conducted for any missing data. A Kolmogorov–Smirnov normality test will be used to examine the normality of the variables. To examine the effects of the intervention, a multilevel regression will be used to compare pain intensity, pain self-efficacy, the use of drug and non-drug methods of pain relief, quality of life, and the knowledge and skills acquired in managing pain situations at baseline (T0), week 8 (T1), and week 16 (T2) if the data are normally distributed. A Generalized Estimating Equation will be used for within-group and between-group comparisons if the data do not follow a normal distribution. A Cohen’s d effect size of the intervention effect will be calculated for all outcomes. A *p*-value of <0.05 will be considered statistically significant. As for a cluster RCT analysis, it is suggested that both a multilevel regression and a generalized estimating equation be used, as they are capable of handling clustered data. Observations from the same participant will fall into a level, and participants from the same NEC will fall into a level, so that both within-subject correlations and intra-cluster correlations can be accounted for.

A qualitative analysis of the contents of the interview data and the WhatsApp text messages will be conducted. The tape-recorded interview data will be transcribed by RA1 and cross-checked for accuracy by the PI or one CI. Finalized transcripts will then be independently coded by two CIs, and important manifest contents and latent meanings in the data will be identified. To achieve consistency and agreement on the meaning of the data, the research team will compare, discuss and agree on codes, and then combine them with verbatim data to form categories/subcategories describing the older adults’ and caregivers’ experiences and perceptions of the benefits and difficulties of participating in the DPM.

For WhatsApp text messages, responses to reminders, questions, and discussions will be organized into themes. A set of categories/subcategories with supporting text message data will be generated to describe the strengths and limitations of the DPM and further improvements that could be made to the DPM.

## 9. Data Management

The research team will be responsible for managing the data. The data that are collected will be locked in a cabinet with a passcode. Only the members of the research team will have the passcode to the cabinet, allowing them access to the data. The confidentiality of the data will be ensured by the research team before, during, and after the study. No personal information will be disclosed.

## 10. Protocol Amendments

The research team will carry out a review to determine if the protocol needs to be revised. The amended protocol will be sent to the Research Ethics Committee of the Hong Kong Metropolitan University, and the participants will be informed when necessary.

## 11. Dissemination Plans

The results of the study will be presented to the public through publications in academic journals and presentations in conferences. Authorship will be determined by the contributions of the research team members.

## 12. Conclusions

A fifth wave of COVID-19 infections occurred in early January 2022, leading to an almost complete cessation of activities. Primary and secondary schools were closed; dine-in services were banned from 6 p.m. to 4:50 a.m. the next day; the number of diners per table was capped at two; many types of specified premises were shut down, including bars, pubs, nightclubs, fitness centers, theme parks, museums, party rooms, event and performance venues, and cinemas; all local tours were suspended; public hospital and nursing home visits were halted; all NECs were closed; and no activities could be carried out [28].

The fifth wave of COVID-19 infections had been subsiding at the time of the writing of this protocol (12 May 2022). The NECs have been gradually resuming their services, and their members (i.e., older adults) are gradually coming back to take part in various activities. Therefore, we are in the process of contacting NECs to carry out the DPM. Given the uncertainty of COVID-19, we are working with the center in charge and considering using zoom for all sessions as a salvage technique should another wave of COVID-19 occur and affect our subject recruitment. We have already secured funding from the Health & Medical Fund, and research assistants have been employed. The team has to be very conscious about maintaining social distancing and following strict infection-control practices for center-based activities. However, we may yet have to consider using Zoom to replace center-based activities or set up smaller groups to avoid large crowds to prevent the possibility of contracting COVID-19.

## Figures and Tables

**Table 1:** Schedule of enrolment, interventions, and assessments of DPM.

	Study Period				
	Enrolment	Allocation	Post-Allocation		
Timepoint	*Pre-Randomization*	*T0*	*Baseline (T0)*	*Week 8 (T1)*	*Week 16 (T2)*
ENROLMENT:					
Eligibility screen	X				
Informed consent	X				
Allocation		X			
INTERVENTIONS:					
*[Experimental Group: Dyadic pain management program]*			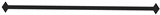		
*[Control group]*					
ASSESSMENTS:					
*[Pain intensity]*			X	X	X
*[Pain self-efficacy]*			X	X	X
*[Perceived quality of life]*			X	X	X
*[Psychological health: Depression, anxiety, stress]*			X	X	X
*[Caregiver Burden Inventory]*			X	X	X
*[Field evaluations]*			X	X	
*[Knowledge and skills acquired in managing pain situations for older adults]*			X	X	X
*[Semi-structured interviews for older adults and their caregivers]*				X	

## Data Availability

Not applicable.
